# Structural Breakdown of Collagen Type I Elastin Blend Polymerization

**DOI:** 10.3390/polym14204434

**Published:** 2022-10-20

**Authors:** Nils Wilharm, Tony Fischer, Alexander Hayn, Stefan G. Mayr

**Affiliations:** 1 Leibniz-Institut für Oberflächenmodifizierung e.V. (IOM), Permoserstr. 15, 04318 Leipzig, Germany; 2 Division of Surface Physics, Department of Physics and Earth Sciences, Leipzig University, Linnéstraße 5, 04103 Leipzig, Germany; 3 Biological Physics Division, Department of Physics and Earth Sciences, Leipzig University, Linnéstraße 5, 04103 Leipzig, Germany; 4 Division of Hepatology, Department of Medicine II, Leipzig University Medical Center, 04103 Leipzig, Germany

**Keywords:** elastin, collagen, polymerization, fiber formation

## Abstract

Biopolymer blends are advantageous materials with novel properties that may show performances way beyond their individual constituents. Collagen elastin hybrid gels are a new representative of such materials as they employ elastin’s thermo switching behavior in the physiological temperature regime. Although recent studies highlight the potential applications of such systems, little is known about the interaction of collagen and elastin fibers during polymerization. In fact, the final network structure is predetermined in the early and mostly arbitrary association of the fibers. We investigated type I collagen polymerized with bovine neck ligament elastin with up to 33.3 weight percent elastin and showed, by using a plate reader, zeta potential and laser scanning microscopy (LSM) experiments, that elastin fibers bind in a lateral manner to collagen fibers. Our plate reader experiments revealed an elastin concentration-dependent increase in the polymerization rate, although the rate increase was greatest at intermediate elastin concentrations. As elastin does not significantly change the structural metrics pore size, fiber thickness or 2D anisotropy of the final gel, we are confident to conclude that elastin is incorporated homogeneously into the collagen fibers.

## 1. Introduction

Thermoresponsive hydrogels find widespread applications in medicine where synthetic and protein-based hydrogels are described. The potential applications include drug delivery, tissue engineering or the separation of bio molecules [1,2,3,4]. For example, an elastin-like polypeptide sequence, attached to graphene, with a high switching rate was designed, which revealed shrinking/bending upon irradiation with NIR (near infrared) light [5]. Examples of drug delivery have been presented by various groups, such as when an elastin-like peptide (ELP) solution is loaded with an anti-tumor drug. After injection into a tumor, the ELP coacervates due to the body temperature and forms a depot from which the drug is released over some time [6]. Similarly, the loading of ELPs with bone morphogenetic protein was described to enhance mineralization [7]. In another application, ELPs were combined with chitosan to form a multilayer system, which changes its wettability state when heated above 50 °C [8]. This system can assist in fine tuning cellular adhesion.

The temperature-induced contraction has an identical root for synthetic as well as protein-based polymers. All these polymers exhibit a lower critical solution temperature (LCST) upon which they become insoluble. The reason for this behavior is the imbalance between hydrophilic–hydrophobic interactions between a polymer and a solvent. Hydrophobic segments along a polymer chain can reduce their solvent-accessible surface area upon an increased temperature by aggregation, which exerts a pulling force on the non-contracting network segments. In fact, the driving force for the contraction is the entropy gain for the solvent molecules. Water molecules around hydrophobic segments are highly ordered but with an increasing temperature this order is disrupted and the hydrophobic segments can associate and fold. The contraction is then actually induced when the entropy gain by the released water molecules is greater than the enthalpy gain by water binding to the polymer [9]. Poly(N-isopropylacrylamide) (PNIPAM) is one of the most investigated thermoresponsive polymers as its transition temperature is relatively insensitive to environmental conditions and is in the physiological regime (~32 °C) [10]. However, PNIPAM polymers have been shown to reduce cell viability for different polymerization types as well as different cell types [11]. Elastin is, therefore, a prime candidate for bio compatible thermoresponsive hydrogels as it is composed of alternating hydrophilic and hydrophobic segments and has already been shown to exhibit a LCST [12]. Recently, a collagen elastin thermoactuator with a tunable transition temperature in the physiological temperature regime was designed [13]. It was demonstrated that the incorporation of elastin from bovine neck ligament into a 2 mg/mL type I collagen gel resulted in a reversible thermoswitchable system with a transition temperature in the physiological temperature regime. As the system showed a temperature-induced phase transition like a volume contraction, it was argued that this process can be described by Euler buckling, which refers to the buckling of a rod under an axial critical load. In fact, two cases are possible when collagen and elastin are polymerized: the formation of individual fibers between collagen fibers (“perpendicular”) and the incorporation of elastin monomers in a parallel manner (“lateral”) into a collagen fiber. Although a lateral fiber alignment seemed likely as the buckling behavior was observed, convincing experiments have been lacking so far. We now present insights into the polymerization features of an elastin collagen hydrogel with significant evidence that elastin is laterally incorporated into the collagen fiber. Both elastin and collagen are the main components of connective tissue and exhibit distinct features. While elastin is structurally heterogeneous as the hydrophilic segments contain some α-helix and the hydrophobic segments are mostly of a random coil design, collagen is relatively homogeneous as three single peptide chains form a collagen triple helix [12,14]. The elastin is expressed in the endoplasmic reticulum, transported outside of the cell and then bound to micro fibrils, where, by a complex mechanism involving the crosslinking of lysine residues, an elastin fiber is formed with elastin on the inside and several micro fibrillar proteins on the outside [15]. Collagen fibrils are formed by an association of collagen triple helical monomers via an enzymatic crosslinking of lysine residues, and several fibrils then associate into fibers; however, our preparation steps were devoid of any crosslinking steps, so that the interaction between the collagen and elastin were dominated by an electrostatic interaction [16].

This work aims to present evidence that the structural metrics pore size and fiber diameter of a type I collagen hydrogel are not significantly changed upon the addition of bovine elastin. As we have found evidence supporting our thesis, we conclude that elastin monomers attach in a parallel fashion to the collagen fibers. This agrees with our own earlier studies with circular dichroism experiments, where we saw a systematic decrease in the helical structures in collagen and elastin after polymerization [13].

## 2. Experiments

### 2.1. Hydrogel Preparation

Collagen hydrogels for all experiments in this study were prepared using the same protocol as described before [13]. The basis for all the hydrogels was a mixture of collagen I monomers from rat tail (collagen R, 0.4% solution and Cat. No. 47256.01; SERVA Electrophoresis, Heidelberg, Germany) and bovine skin (collagen G, 0.4% solution and Cat. No. L 7213; Biochrom, Berlin, Germany) in a mass fraction of 1:2, respectively. To initiate the polymerization of the monomer mixture solution, a 1 M phosphate buffered solution containing disodium hydrogen phosphate (Cat. No. 71636, Merck KGaA, Darmstadt, Germany), sodium dihydrogen phosphate (Cat. No. 71507, Merck KGaA, Darmstadt, Germany) and ultrapure water was added to produce a final pH value of 7.5, ionic strength of 0.7, and a phosphate concentration of 400 mM. To produce the collagen–elastin hydrogels, appropriate amounts of elastin powder (elastin, Cat. No. 6527, Merck KGaA, Darmstadt, Germany) were added to the buffer solution prior to the polymerization. All solutions were kept on ice. The polymerization of the final solutions was initialized by placing the samples in an incubator at 37 °C.

### 2.2. UV/VIS Plate Reader Experiments

The experiments were performed on a TECAN infinite^®^ 200 plate reader (absorbance mode, 405 nm, target temperature 37 °C, 25 flashes, and a sampling rate of 1/min; TECAN Trading AG, Männedorf, Switzerland) using flat bottom 96-well plates (Carl-Roth GmbH, Karlsruhe, Germany) for the sample preparation and measurement. For a single experiment, three solutions were prepared directly before the measurement, namely, (I) 1.2 mL of a 2 mg/mL collagen solution (0.4 mL R, 0.8 mL G, each 4 mg/mL of stock solution), (II) 1.2 mL of a 2 mg/mL collagen solution (same as above) with 0.6 mg of elastin and (III) 1.2 mL of a 2 mg/mL collagen solution (same as above) with 1.2 mg of elastin. A 200 µL amount of the final solution was filled in each well, resulting in 6 wells of a 96-well plate per condition and 18 wells for all three conditions. The remaining 78 wells were filled with distilled water to ensure high humidity during the polymerization and to counteract the dehydration of the samples.

### 2.3. Zeta Potential

A 6 mg amount of elastin was dissolved in a 3 mL phosphate buffer (pH 7.5, 400 mM) at 4 °C. Additionally, 3 mL of collagen (1 mL R and 2 mL G, each 4 mg/mL stock solution) was mixed with 3 mL of the phosphate buffer (pH 7.5, 400 mM) at 4 °C. Both solutions were subjected to zeta potential measurements on a NanoZS (Malvern) zetasizer with a backscatter optical arrangement (173°). Each condition was measured three times by preparing a fresh solution each time. Each sample (1 mL) was left for 120 s in the instrument, which was precooled at 4 °C, to reach the thermal equilibrium. Each sample was measured eight times with twenty runs and a 30 s delay between the measurements.

### 2.4. Pore size and Fiber Diameter

The collagen and collagen–elastin samples were prepared as described above. A 200 µL amount of the ice-cooled solution was placed in each well of a cooled 24-well µ-plate (µ-Plate 24 Well ibiTreat; Cat. No. 82406; ibidi, Gräfelfing, Germany). Subsequently, the 24-well plate was placed in an incubator at 37 °C to start the polymerization for 2 h. The polymerized hydrogels were washed three times using PBS and fluorescently stained by applying 20µg/mL of 5(6)-Carboxytetramethylrhodamine N-succinimidylester (TAMRA-SE; Cat. No. 21955; Merck KGaA, Darmstadt, Germany) overnight and subsequently washed three times using the PBS. Using an LSM microscope (TCS SP8; Leica, Wetzlar, Germany), three-dimensional image cubes of the fluorescence signal of the TAMRA-SE using a 561 nm excitation laser and HC PL APO CS2 40x/1.10 water immersion objective were recorded. The pore size and fiber diameter were determined as published previously [17,18]. To compensate for the apparent collagen–elastin clusters that would disturb a fiber diameter determination, a custom-built cluster deletion algorithm was used to solely measure the actual fiber diameter sizes.

### 2.5. Directionality

The above obtained images were also used to quantify the directionality and its standard deviation of the network. The imageJ plugin, “Directionality”, with the “Fourier components” method was used. A total of 70 planes of each of 10 different random positions for both gels (collagen and collagen–elastin) were summarized into one image which was then analyzed. The clusters in the collagen–elastin samples were removed prior to analysis using the same, custom-built cluster deletion algorithm as described above.

### 2.6. Elastin Influence on the Network Structure

To investigate the influence of elastin polymerization on the final hydrogel structure, we used a Col-F collagen binding reagent (Col-F; Cat. No. 6346; ImmunoChemistry Technologies, Bloomington, MN, USA) and a collagen I antibody (Immunotag™ Collagen I Polyclonal Antibody; Cat. No. #ITT5769; G-Biosciences, St. Louis, MO, USA). The collagen–elastin hydrogels were prepared in 24-well µ-plates as described above.

For the collagen I antibody staining, the samples were incubated with 5% BSA solved in PBS for 30 min at room temperature—with aspirate goat serum, and incubate sections with primary antibody (ITT5769) in PBS overnight at 4 °C or 1 h at 37 °C; 3 × 1:1000 (600 µL/well). The samples were washed three times with PBS for 5 min each.

For the Col-F staining, the samples were incubated with 3% BSA solved in PBS for 30 min at room temperature—with aspirate goat serum, and incubate sections with primary antibody (ITT5769) in PBS overnight at 4 °C or 1 h at 37 °C; 3 × 1:200 (300 µL/well). The samples were washed three times with PBS for 5 min each.

Three-dimensional images were recorded using an LSM microscope (TCS SP8; Leica, Wetzlar, Germany) with a 63×/1.20 HC PL APO CS2 water immersion objective and a 488 nm (Col-F) and 561 nm (collagen antibody) excitation laser, respectively. The final image dimensions were 100 µm by 100 µm in x-y and a roughly 30 µm to 50µm z dimension.

### 2.7. Live Polymerization

The samples were prepared as described above. A 1 mL amount of the cooled solution was placed in a well of a pre-cooled 24-well µ-plate and then placed in a LSM microscope with an incubation chamber (TCS SP8; Leica, Wetzlar, Germany) at 37 °C and 100% relative humidity. Using a HC PL APO CS2 40×/1.10 water immersion objective and a 561 nm laser in the reflection mode, a 1 h recording of the polymerization process and hydrogel network formation was observed and recorded as live imaging videos. The videos had an image size of 1024 × 1024 px with a frame-rate of 1 fps.

### 2.8. Statistical Methods

The employed statistical methods included the mean, median, standard deviation and a box plot as well as a Mann–Whitney-U test. The methods are named at the relevant position.

## 3. Results

### 3.1. Plate Reader

**Figure 1:**
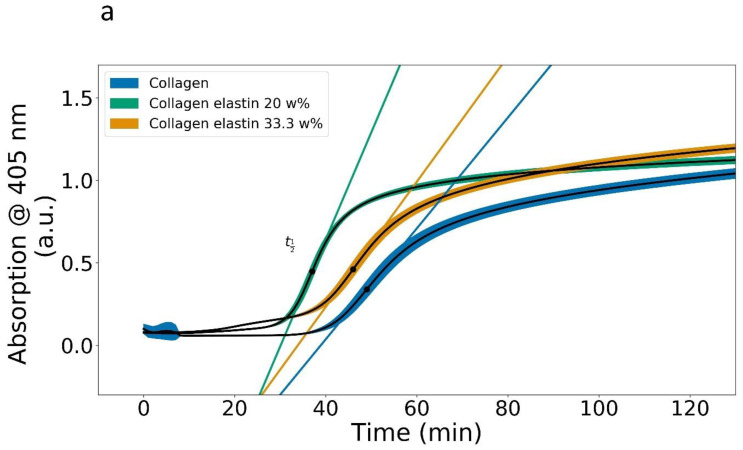

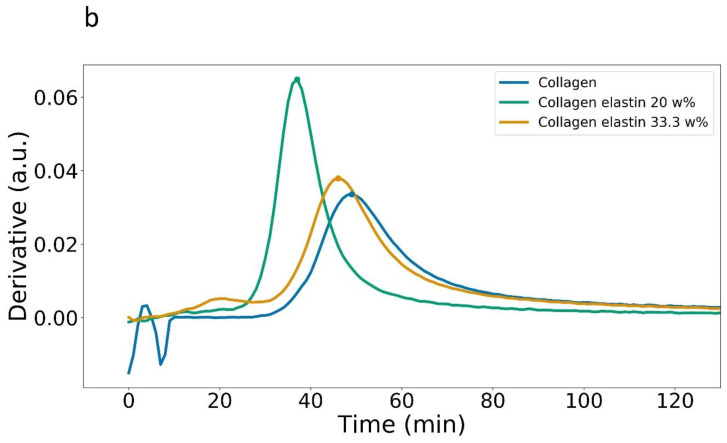
(**a**) Mean polymerization curves at 37 °C for a 2 mg/mL collagen solution as well as two collagen–elastin solutions containing 20 w% (0.6 mg/mL elastin) and 33.3 w% (1.2 mg/mL elastin), respectively. (**b**) Derivative of the mean of the curves in (**a**). Six wells were recorded per sample and the color-coded curves in figure (**a**) denote one standard deviation. Supplementary Figure S3 shows the extended curves. The random spikes in the beginning of the collagen curve result from water condensation and evaporation under the well plate cover.

Figure 1 displays the polymerization curves of a collagen solution and two collagen elastin solutions at 37 °C. The heating curve of the collagen is in strong agreement with the literature data, as it highlights the onset of clouding after 30 min as well as no significant changes in the turbidity after 2 h [19]. The clouding curve is generally associated with fiber formation which increasingly contributes to light scattering. The addition of elastin then introduces several features into the polymerization process. The most striking feature is that the final absorption (>2 h) was only slightly increased, although 25% or 30%, respectively, should be expected as this is the net increase in the biomass for each sample. This is a strong indication that the alignment of elastin and collagen monomers must occur in a lateral manner, as the opposite case of a perpendicular alignment would contribute to light absorption and scattering. The small increase in absorption, at times >2 h, might be a consequence of an elevated fiber thickness as elastin monomers attach to the collagen triple helix. The lateral addition of elastin is also likely as circular dichroism experiments on elastin–collagen gels have shown that the addition of elastin leads to a reduced PPII (poly-proline II) content, probably due to PPII helix distortion [13]. The addition of low amounts of elastin increases the polymerization rate by a factor of two while the polymerization rate maximum is shifted to an earlier time (37 min instead of 49 min, see Table 1). At these concentrations, the elastin may act as a nucleation center for polymerization. Additional effects which could fasten the assembly might include the burying of hydrophobic domains in collagen but especially in elastin, which has alternating hydrophilic and hydrophobic segments [20,21]. Additionally, a potential entropy gain by a helix distortion, as mirrored in the reduced PPII helix content in collagen after an elastin addition, supports the thesis of a conformation-dependent collagen–elastin interaction [13]. Such an entropy gain by a helix distortion was described for an alpha helix [22]. Taken together, collagen’s and especially elastin’s propensity to bury their hydrophobic domains, as well as a general increase in the monomer concentration, might contribute to an increase in the polymerization rate. In terms of the type of fiber alignment, we argue that hydrophobic burying implies a parallel alignment, as in the otherwise perpendicular type no significant burying can take place.

A further addition of elastin, however, decreased the polymerization rate. This was unexpected as the polymerization rate is always proportional to the monomer concentration. Thereby, at relevant concentrations, elastin can be viewed as a perturbation towards polymerization as it may interfere with the proper alignment of collagen triple helical monomers. A fingerprint of this feature was the additional absorption shoulder at 20 min which probably signified a second polymerization process introduced by the elastin. This shoulder is believed to originate from the formation of elastin–collagen clusters which form at elevated elastin concentrations. This is a likely process, as elastin to collagen ratios of more than 0.22 will exceed elastin–collagen equimolarity. In fact, based on the molar masses of collagen (300,000 Da) and elastin monomers (ca. 67,000 Da), 0.5 mg/mL of elastin is sufficient to accommodate 2 mg/mL of collagen in an equimolar manner [23,24]. The plate reader experiments fell well within this consideration, as they showed that 0.6 mg/mL of elastin did not lead to an additional clustering peak at 20 min, while the 1.2 mg/mL sample did so; therefore, the upper limit for an elastin addition seems to lie between these values. As the absorption value in the 33.3 w% curve of the 20 min peak was much smaller than the maximum absorption, it can be argued that most of the biomass was polymerized into the gel. Another interpretation may be that the shoulder at 20 min signified clouding by elastin coacervation, a well-known effect which describes the heat-induced elastin aggregation by an association of hydrophobic elastin segments. However, elastin coacervation is quite fast and usually complete after several minutes; therefore, we can exclude this effect here [25]. It is important to note that a similar experiment was performed by Vazquez-Portalatin et al. by also using collagen type I and bovine neck ligament elastin. They similarly recorded the clouding of elastin–collagen solutions for several elastin–collagen ratios [26]. Opposed to our experiments, they observed an overall increase in the polymerization rate and a shift in the polymerization start to earlier times with an increasing elastin proportion; however, the maximum polymerization rate was around 21 min, which was twice as fast as our observation of around 40 min. Additionally, the turbidity-dependence on the elastin concentration was much lower than in our experiments. This might not only have to do with the fact that they used only rat tail collagen (R collagen), another wavelength (313 nm) and PBS (phosphate-buffered saline) instead of a phosphate buffer. In fact, they used comparable elastin–collagen ratios but with a 1:10 dilution. This gives credit to our above claim of a saturative process during polymerization. Obviously, in our experiments, the addition of elastin at elevated concentrations seemed to induce a second polymerization process apart from the “classical” polymerization which we introduce as a cluster formation, probably because the monomers met more often which also increased the chance that the monomers met without being optimally aligned in the gel. These clusters grew on their own without participating in the classical polymerization. In fact, Paderi et. al. discuss how a perpendicular chain alignment can inhibit collagen polymerization which may, in our case, have been the nucleation center for the cluster formation [27].

### 3.2. Videos of Polymerization

Videos S1–S3 (supplement) show the fiber formation of a collagen solution and two elastin–collagen solutions, while Video S4 (supplement) shows a comparison video. Video S1 is characterized by early and quick flashes of fibers and nodes which resulted from their diffusion through the focal plane. Small fibers and nodes could be observed as early as five minutes after combining both solutions (the collagen stock and buffer). This was contrasted to the plate reader experiments where no significant changes in the absorbance were observed before 25 min. This was because the plate reader measures absorbance which is quite small for small particles, so that only sufficiently large particles or fibers can contribute to the absorbance. The videos emphasize that the fibers assembled rather quickly while they were still subjected to convection, i.e., liquid flow. The onset of polymerization was characterized by a fiber flow velocity reduction which came to a complete stop as soon as sufficiently large enough fibers had come into contact. The fiber growth occurred in the early stages end to end and was then followed by a fiber thickening, which is in line with the literature claims that axial growth is much faster than lateral growth [28]. The sequence of the axial followed by the lateral fiber growth was retained when the elastin was added, implying that the elastin did not significantly interfere with the fiber assembly process in terms of the network structure. Moreover, when the polymerization sequence of the collagen–elastin solution was identical to the one of the pure collagen polymerization sequence, then the elastin must have been homogeneously incorporated into the collagen system, i.e., laterally. It was further obvious, that the elastin-containing networks polymerized earlier, which was in good agreement with the plate reader experiments. Consequently, elastin seemed to facilitate polymerization as described above, although this effect was concentration-dependent. The maturing collagen network was still drifting through the focal plane as seen in the appearance and disappearance of fibers and nodes. This implies that the network was subjected to density fluctuations during the polymerization. This was contrasted to the elastin containing networks, which did not drift through the focal plane. This might relate to our observations, namely, that the elastin-containing gels appeared to stick to the walls of the petri dish. This effect might limit the z-drift. A final observation was that the elastin-containing networks contained some clusters which were more prominent in the high-elastin concentration sample. Video S4 shows quite nicely how these clusters disappeared after around 30 min. We believe that the clusters sunk either to the bottom of the gel or were bound randomly to the existing fibers, although we could not observe such diffusion to the fibers. We further argue that the presence of these clusters coincided with the presence of the elevated absorbance around 20 min in the elastin-containing turbidity curves (Figure 1); however, further experiments are required to understand the interaction between the elastin and collagen R and G. This question bears some importance, as G collagen is more closely related to the formation of nodes than R collagen [19]. In fact, further applications might demand answering the question of whether elastin is also present in the nodes as a local matrix stiffness can guide the cell migration [19].

### 3.3. Zeta Potential

**Figure 2:**
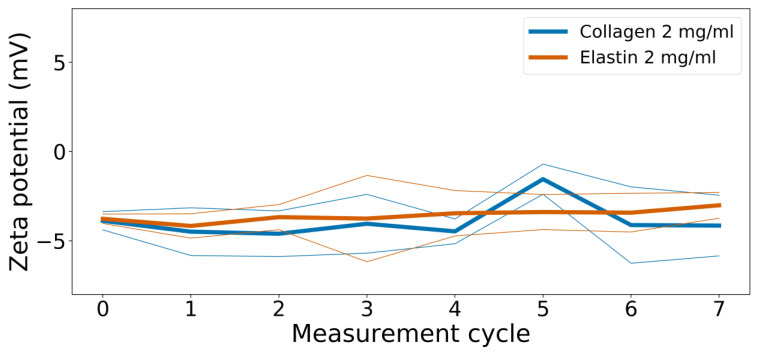
Zeta size measurements of a collagen (2 mg/mL) and an elastin solution (2 mg/mL) in a phosphate buffer at pH 7.5 and 4 °C. Incremented mean (thick lines) and one standard deviation (thin lines) are shown. As data points along the curves have slightly varying total run times from 7 to 10 min (due to variations in temperature regulation by the device), the data points were averaged accordingly and plotted over the increment. Original data can be found in supporting Figure S4.

The zeta potential measurements of the individual collagen and elastin solutions in the phosphate buffer at pH = 7.5 and 4 °C revealed that all solutions exhibited a zeta potential around −4 mV (Figure 2). Values in this range are optimal for aggregation as values smaller than ± 30 mV are considered to induce aggregation [29]. Although biopolymers such as collagen and elastin have plenty of ionizable groups, zeta potential values around zero indicate a low degree of ionization. This low potential, as described above, favors monomer aggregation in any way, including laterally, as the resulting hydration shell will be small at these values so that the repulsion will effectively play no role. In fact, both collagen assembly and elastin assembly (coacervation) are endothermic and entropy driven at 37 °C, while the loss of an ordered hydration shell is the largest contribution to entropy gain [25,30]. However, a decrease in Gibbs energy is roughly twice as much in collagen than it is in elastin, implying that collagen can more easily lose its hydration shell. Moreover, although the thermodynamics for elastin relate to the effect of coacervation, we did not see this effect in the plate reader experiments, where no early clouding could be detected. Collagen and elastin monomers could, therefore, align in a parallel manner before a temperature increase shifts the Gibbs energy change from positive to negative such that, after a loss of the respective hydration shell, the collagen–elastin association is more favorable than an elastin–elastin association (coacervation). Elsewhere, the lateral merging of a hydration shell of peptides has been described which opens up the possibility of a multistep mechanism of an early elastin–collagen interaction [31]. It is also noted that the employed collagen was already in its triple helical state so that the elastin should not have interfered with the triple helix formation; however, the data of Wilharm et. al. show that the circular dichroism of collagen–elastin is not an ideal superposition for each component and that it lacks some PPII content [13]. This shows how the presence of elastin might impact the collagen helix anyway, probably due to the destabilization of the intricate H-bond equilibrium in collagen, probably in a lateral manner.

### 3.4. Directionality

**Figure 3:**
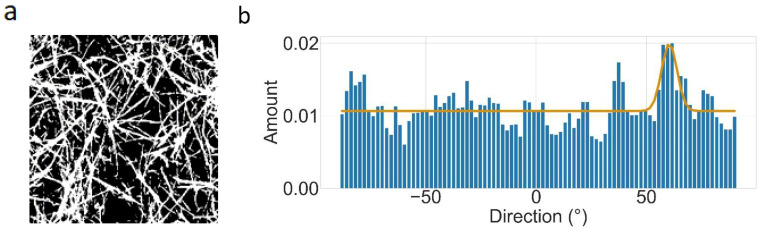
(**a**) Exemplary skeletonized image of a 2 mg/mL collagen network. (**b**): Angle distribution of (**a**). The degree values are given in the mathematical sense, i.e., 0° is pointing to the right. The fit is a Gaussian function, provided by ImageJ.

**Figure 4:**
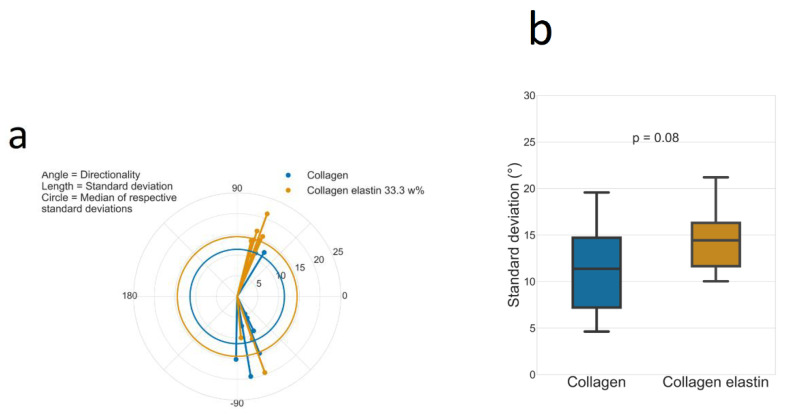
(**a**) Polar plot of the 2D anisotropy analysis of each 10 random positions in one 2 mg/mL collagen network and one collagen–elastin (33.3 w% elastin) network. The coordinates refer to the determined angle (polar angle) and the standard deviation (radial length) while the circles refer to the median values of the distributions of the standard deviations, i.e., elastin increases the standard deviation of the network and, thereby, the 2D anisotropy. (**b**) Comparison between the standard deviation of the angle distribution of the samples already displayed in Figure 4. Significance was tested with the Mann–Whitney U test. This standard deviation is referred to as “2D anisotropy”.

Figure 3 shows the 2D anisotropy for an exemplary network, while Figure 4 compares the 2D anisotropy of a collagen and a collagen–elastin network. The sections of all samples show two preferred directionalities, one around 65° and another around −80°. The directions seem to be inversely populated by collagen and elastin–collagen. Regarding the origin of this preferred orientation, one explanation might be the gelation condition. In fact, the gels were gelled within an incubator placed on a microscope. The incoming air and humidity induced a mild current which might have oriented the fibers accordingly. This is also visible in the Videos S1–S4 where the material is drifting until the polymerization starts and the flow is restricted. Although this drift was unavoidable when using this experimental approach, this technique was used to specifically prepare oriented gels [32]. While the addition of elastin did not change the network directionality, the standard deviation of the angle distribution might have been slightly increased (Figure 4). This direct comparison between the respective standard deviations across the angle distributions of all 10 position reveals a minor significant difference as the Mann–Whitney U test was *p* = 0.08, which was larger than the generally accepted threshold of 0.05. Under the assumption of this threshold, both distributions would not originate from one set of data, i.e., the addition of elastin would lead to a flattened angle distribution. It can be concluded that elastin’s presence interferes with the formation of larger, similarly oriented domains. Mostaço-Guidolin et al. have found the interesting observation that a similar orientation of collagen and elastin fibers in the arterial wall of rabbits was greatest when they were middle-aged and lowest when they were young or old [33]. In the context of our experiments, this might imply that an elastin addition creates less mature networks as it seems to slightly interfere with a proper alignment of collagen fibers. The above-described plate reader experiments support this claim, as they showed an elastin concentration-dependent increase in the polymerization rate. A faster rate means less time for the monomers to perfectly align, such that stacking irregularities can occur. This was already discussed earlier, where a faster rate was suspected to contribute also to the cluster formation. Nonetheless, our analysis of the 2D anisotropy in the collagen and elastin–collagen networks could not conclusively portray a difference in the 2D anisotropy, as the *p*-value was just slightly larger than 0.05, which supports our claim of a lateral elastin–collagen alignment. In fact, a predominantly perpendicular alignment of the collagen and elastin fibers should significantly increase the standard deviation of the angle distribution. Additionally, the above-mentioned bubbles were removed prior to the 2D anisotropy analysis so that the observed potential increase in the standard deviation might as well have originated from this preprocess, implying that there was no real difference at all between the collagen and elastin in terms of the 2D anisotropy. The above arguments are in line with the narrative that if 2D anisotropy as a network metric does not significantly change upon an elastin addition, then the structure cannot be changed, i.e., elastin is incorporated mostly homogeneously into collagen.

### 3.5. Laser Scanning Microscopy (LSM)

**Figure 5:**
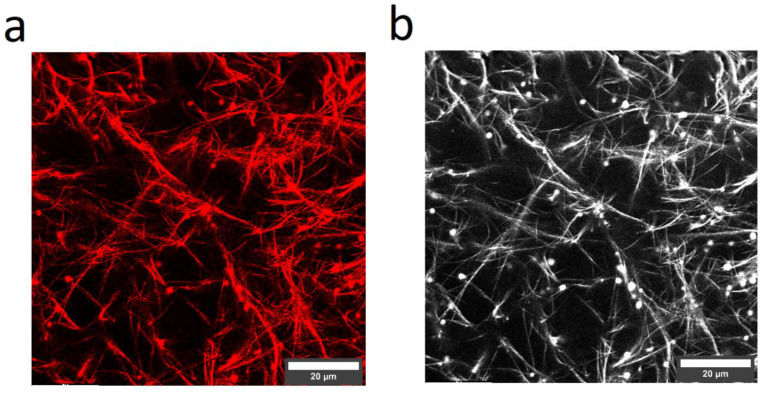
Fluorescence images of a 33.3 w% elastin–collagen gel. (**a**) collagen type I antibody and (**b**) Col-F. The dots in each image are most likely clustered elastin–collagen monomers.

LSM recordings of a collagen–elastin hydrogel with primary collagen antibody staining support the above claims of lateral collagen–elastin polymerization (Figure 5). The left image (Figure 5a) represents an exemplary primary collagen antibody-stained sample. In total, images from seven random positions were recorded which all displayed the discussed features.

The right image (Figure 5b) shows all the network features (collagen + elastin). A comparison with the primary collagen antibody-stained image reveals identical features in both images while any observable differences must be attributed to the brightness thresholding of the image software. The left image contains the well-known features of the R + G collagen mixture, namely, the nodes and fibers, while the right image does not convey any additional structural features; therefore, a lateral alignment of the elastin and collagen monomers appears likely. This is further plausible, given the architecture of the elastic fiber under physiological conditions. Elastin is synthesized in the endoplasmic reticulum and then transported outside of a cell by binding to an elastin-binding protein. Upon binding of this protein to the galactosugars of micro fibrils outside of a cell, elastin is released from the elastin-binding protein and interacts then with the microfibrils. Elastin is then incorporated in a complex way into the microfibrils resulting finally in a fiber which contains elastin on the inside and a microfibrillar shell outside [15]. Basically, elastin needs a scaffold to be deposited on and several proteins of the fibrillin class as well as MAGP-1 where it is shown to interact with elastin [34]. Furthermore, although the elastin–fibrillin interaction is very complex, it has been shown that elastin binds to a glycine and proline-rich region in fibrillin-2 [35]. Consequently, some homology to collagen is given, which lends credibility to the elastin–collagen interaction as seen in the above described LSM recordings; however, other proteins such as fibulin-5 are also central to elastic fiber formation [36]. The list of important proteins continues and their non-existence in our system may be a likely explanation for the lack of formation of distinct elastic fibers. This consideration hardens our claim of a lateral, or at least, homogenous incorporation of elastin into collagen fibers, as elastin monomers simply do not experience guidance and as such are subjected to following collagen fibrillogenesis.

The image in Figure 5 contains the clustered collagen and elastin which was already discussed in the section, “plate reader”, where the high elastin concentration sample displayed an absorption peak prior to the main maximum. These clusters are said to contribute to clouding as the early binding of elastin to collagen might form these clusters which, by chance, are not polymerized into the final network. As we can see the clusters also in the “collagen only” channel (Figure 5a), they must have at least contained some collagen, but as the clusters also appeared after the elastin addition, they must have also contained elastin.

**Figure 6:**
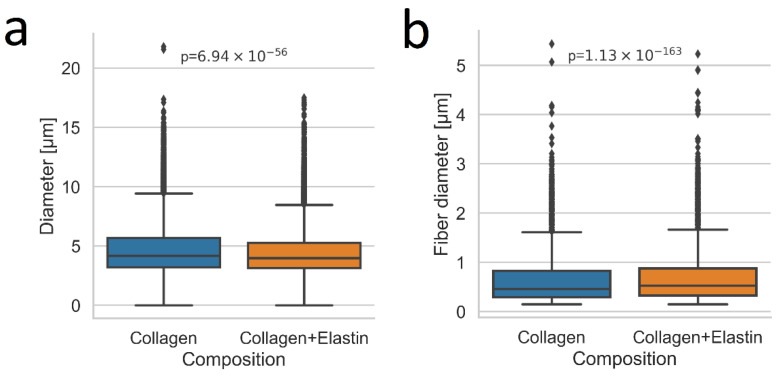
(**a**) Pore diameter of a 2 mg/mL collagen gel and a 33.3 w% elastin collagen gel. (**b**) Fiber diameter of the same gel. Ten positions for each condition were used and 100 planes were summed each prior to analysis. Significance was tested with the Mann–Whitney U test.

**Figure 7:**
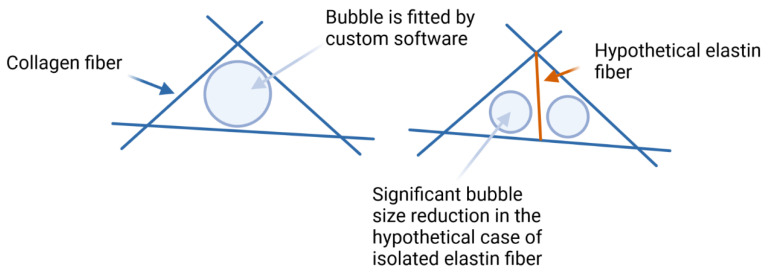
Drawn intersection of collagen fibers (blue lines), which enclose a pore (circle); however, a hypothetical elastin fiber (red line) will divide the pore in two much smaller pores. As we did not see a significant decrease in the pore size, the only other mechanism must be lateral polymerization.

An interesting accordance is seen when the 3D pore size of the networks is compared (Figure 6). The addition of elastin lowered the median pore size by only ~4%. Additionally, the interquartile range was smaller after the elastin addition (0.46 µm for the collagen and 0.32 µm for the collagen–elastin). A likely explanation for this effect is an increase in the fiber diameter because of a lateral fiber alignment between the elastin and collagen chains. The resulting thicker fibers would automatically lead to a decreased pore size when the network architecture remains unchanged, which was shown earlier. Indeed, Figure 6 reveals an increase in the fiber thickness by ~10% which is, again, similar to the percentage changes for the pore size. In fact, other imaginable polymerization types, i.e., a branched fiber alignment, should significantly reduce the median fiber thickness. Consider also the network illustration shown in Figure 7. If the addition of elastin to the network would connect random points along the turquois fibers, the pore size would be halved or at least significantly reduced. This effect must lead to a significant decrease in the median pore size which, presently however, was not observed.

## 4. Discussion

**Figure 8:**
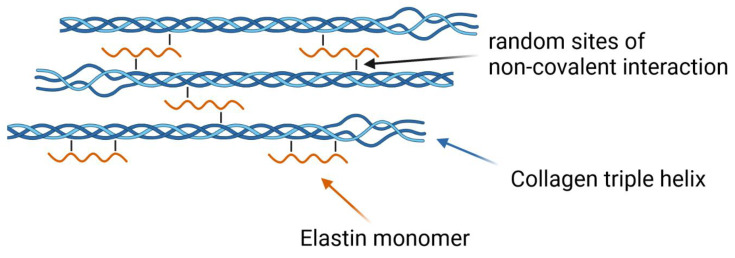
Proposed incorporation of elastin into a collagen fibril (hydrophilic and hydrophobic segments of elastin are not shown). The random coil ends of collagen should signify the suggested interference of elastin with collagen’s secondary structure during polymerization. Elastin monomers are thought to bind to collagen through local H-bonds, van-der-Waals bonds and ionic bonds, although the latter is less likely due to the low zeta potential.

The most remarkable finding within our experiments was that the addition of elastin to a collagen solution at pH 7.5 does neither induce significant changes within the polymerization process nor structural changes within the network later. Initially, we discussed two extremes of a collagen–elastin interaction, namely, perpendicular and lateral polymerization. While we expected a mixed state between these two extremes prior to the experiments, it became quickly clear that the experiments favored the lateral state over the perpendicular and mixed state (Figure 8). This was also initially proposed as we had observed a Euler buckling-like behavior of the hybrid gels under heating in earlier experiments [13]. This phase transition-like behavior can only manifest if elastin conveys a compressive force on collagen fibers in the axial direction. In the opposite case of a perpendicular connection, one would expect a linear decline in the volume with the temperature, as the collagen network would gradually follow the contractive force conveyed by elastin. An homogenous incorporation by a lateral fiber alignment is also likely from another perspective. The persistence length l_p_ of a polymer describes the length over which bending fluctuations are correlated, where a larger value means that the respective polymer is rather inflexible. The literature reports values for collagen of l_p_ = 10 nm to 20 nm and for elastin of l_p_ = 0.3 nm to 0.6 nm [37,38,39]; therefore, elastin monomers are around 30 times more flexible than collagen monomers. Together with only 1/4th of collagen’s mass, it is easily imaginable that elastin monomers attach quickly and in a highly adaptive manner to collagen monomers.

## 5. Conclusions

We showed insights into the polymerization features of elastin–collagen hydrogels. Especially, it was shown that elastin and collagen chains interact in a lateral fashion. This was directly demonstrated with the LSM recordings of collagen and collagen–elastin gels where the collagen was separately stained over the collagen–elastin and further indirectly, as the addition of elastin did not change the structural metrics pore size, fiber thickness or 2D anisotropy. Although we did not quantify changes in the axial and lateral polymerization rate, a visual inspection of the Videos S1–S4 highlights no changes in this polymerization metric after the elastin addition, i.e., the axial fiber growth still starts earlier than for lateral growth; however, the plate reader experiments revealed an elastin concentration-dependent acceleration of the polymerization rate and no signs of elastin coacervation in the presence of collagen. This is a strong sign that a lateral elastin–collagen association precedes the temperature-induced loss of the hydration shell in both, leading to homogenous elastin–collagen hybrid fibers. Further, the zeta potential experiments confirmed a similarly low potential for elastin and collagen, confirming optimal conditions for aggregation. Taken together, the addition of bovine neck ligament elastin to type I collagen solutions accelerated the polymerization rate, although no significant structural changes of the resulting gels could be observed. To generalize our findings, we showed elastin’s propensity to bind to other bio polymers such as collagen in a lateral manner and our findings can help to guide the preparation of other elastin-based bio materials with or without actuatoric application.

## Figures and Tables

**Table 1:** Characteristic values for polymerization. $t_{\frac{1}{2}}$ stands for the time where the derivative of the turbidity curves has the greatest value, i.e., the increase in turbidity is the greatest, while Abs. at t(1/2) (a.u.) stands for the absorption value (turbidity) at the time of $t_{\frac{1}{2}}$.

	Maximum Rate (a.u./Time)	$t_{\frac{1}{2}}$ (min)	Abs. at $t_{\frac{1}{2}}$ (a.u.)	Abs. after 120 min (a.u.)
Collagen, 405 nm	0.033	49	0.34	1.01 ± 0.03
Collagen + 0.6 mg Elastin, 405 nm	0.064	37	0.46	1.11 ± 0.02
Collagen + 1.2 mg Elastin, 405 nm	0.037	46	0.45	1.17 ± 0.03

## Data Availability

Not applicable.

## Supplementary Materials

The following supporting information can be downloaded at: https://www.mdpi.com/article/10.3390/polym14204434/s1, Figure S1. Left: 10 stacked images of a 2 mg/mL collagen type I gel. Right: cluster detection as indicated by black dots. These were ignored for the fiber thickness estimation as shown in Figure S2; Figure S2. Left: exemplary 2 mg/mL collagen gel. Right: fiber thickness estimation. Detection was similarly undertaken for the elastin–collagen after cluster removal as shown in Figure S1; Figure S3. Top: extended polymerization curves. Mean polymerization curves at 37 °C for a 2 mg/mL collagen solution as well as two collagen elastin solutions containing 20 w% (0.6 mg/mL elastin) and 33.3 w% (1.2 mg/mL elastin), respectively. Bottom: derivative of the mean of the top curves. Six wells were recorded per sample and the color-coded curves in the top figure denote one standard deviation; Figure S4. Measured zeta potential values over time. Three samples were analyzed per condition. Video S1: Collagen; Video S2: 20 Elastin + Collagen; Video S3: 33.3 Elastin + Collagen; Video S4: Comparison.
