# Supplementary material for: Structural Breakdown of Collagen Type I Elastin Blend Polymerization
Source: Polymers (Basel). 2022 Oct 20;14(20):4434. doi: 10.3390/polym14204434 (PMC9611167; doi:10.3390/polym14204434)
Supplement: Supplementary file 1 [file polymers-14-04434-s001.zip › polymers-1953508-supplementary.pdf]

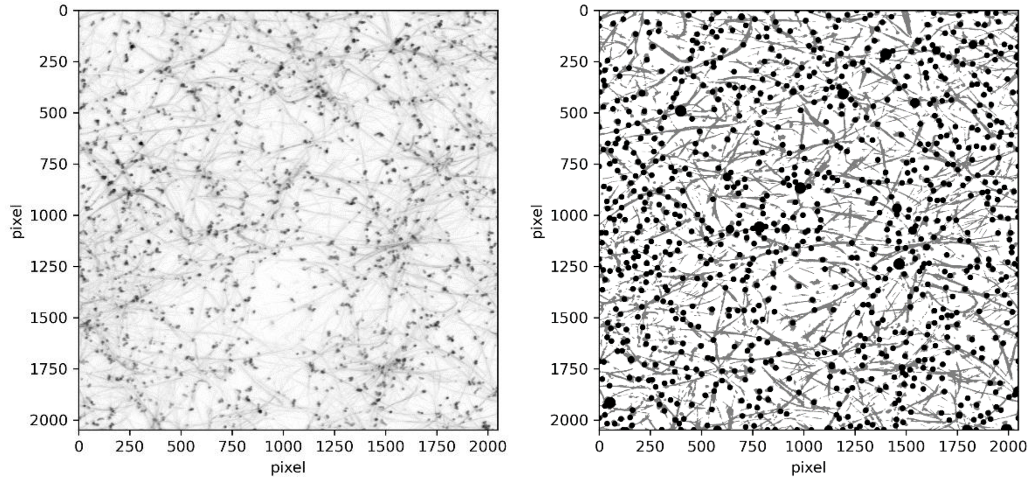

Figure S1. Left: 10 stacked images of a 2 mg/ml collagen type I gel. Right: Cluster detection as indicated by black dots. These were ignored for the fiber thickness estimation as shown in Figure S2.

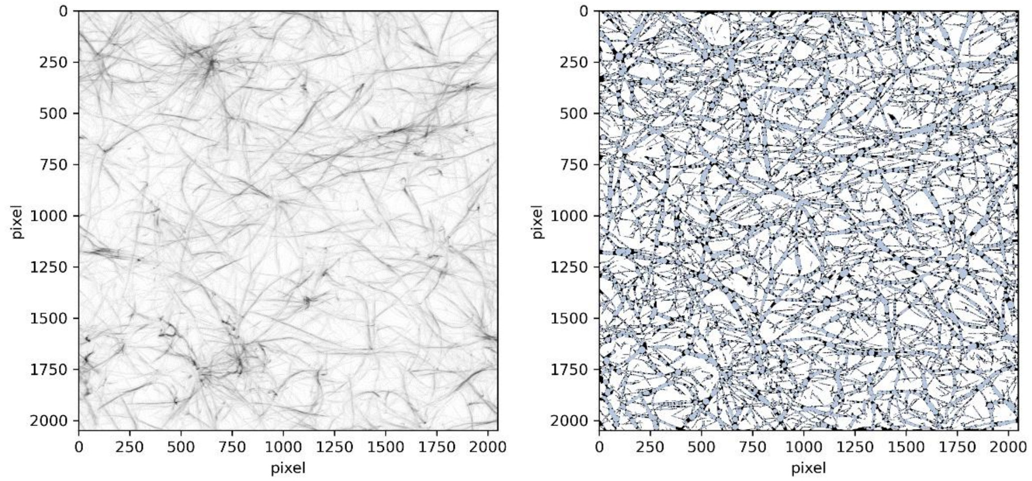

Figure S2. Left: Exemplary 2 mg/ml collagen gel. Right: Fiber thickness estimation. Detection was similarly done for elastin collagen after cluster removal as shown in Figure S1.

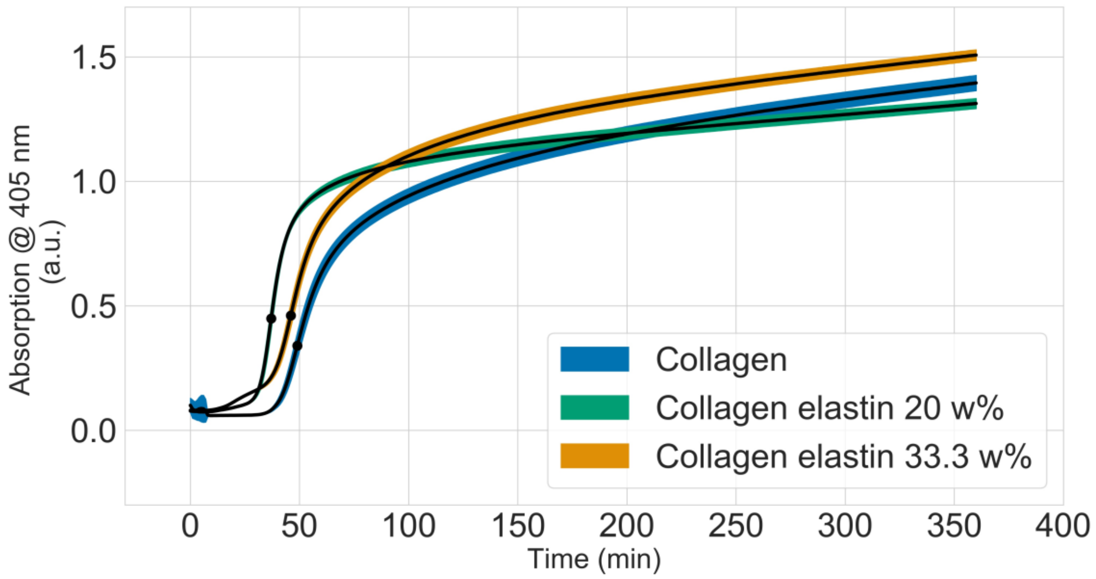

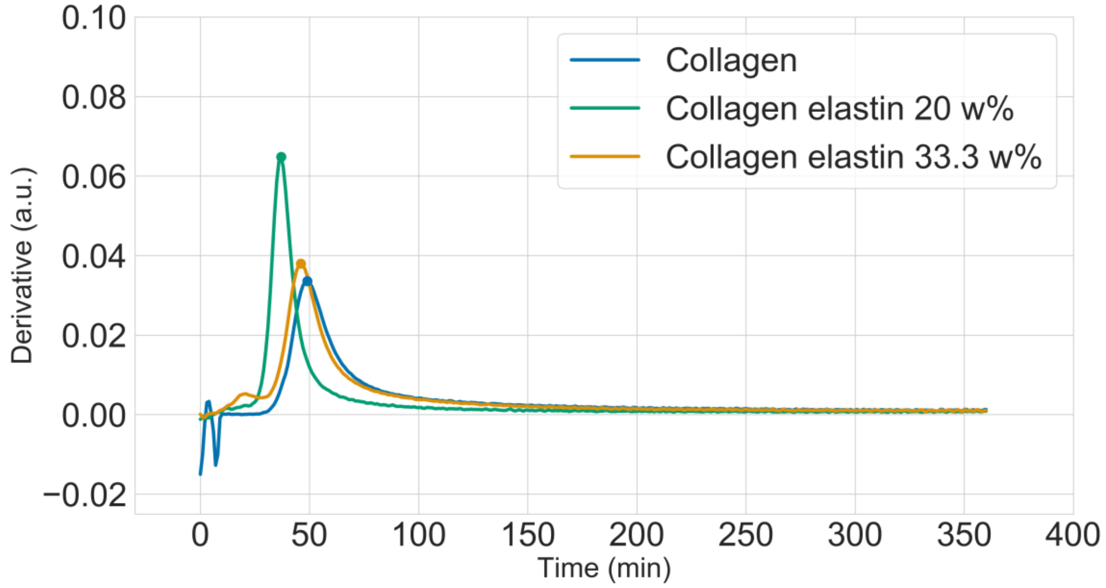

Figure S3. Top: Extended polymerization curves. Mean polymerization curves at 37 °C for a 2 mg/ml collagen solution as well as two collagen elastin solutions containing 20 w% (0.6 mg/ml elastin) and 33.3 w% (1.2 mg/ml elastin) respectively. Bottom: Derivative of the mean of the top curves. Six wells were recorded per sample and the color-coded curves in the top figure denote one standard deviation.

Video S1 - Video of a 2 mg/ml collagen gel during polymerization.

Video S2 - Video of a 2 mg/ml collagen + 0.5 mg/ml elastin gel during polymerization.

Video S3 - Video of a 2 mg/ml collagen + 1.2 mg/ml elastin gel during polymerization.

Video S4 - Comparison of the videos V1 – V3, where V1 is left, V2 is in the middle and V3 is right.

Video Link： https://speicherwolke.uni-leipzig.de/index.php/s/yDa4fEkgS82eTFE

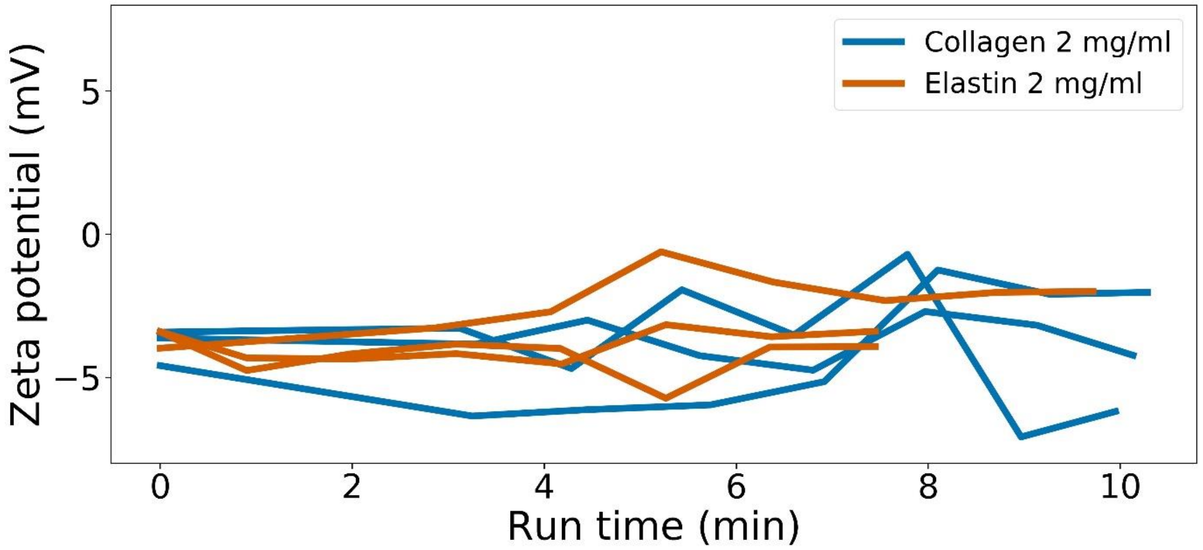

Figure S4. Measured zeta potential values over time. Three samples were analyzed per condition.
